# Heavy Metals Contamination in Shellfish: Benefit-Risk Evaluation in Central Italy

**DOI:** 10.3390/foods9111720

**Published:** 2020-11-23

**Authors:** Francesca Barchiesi, Raffaella Branciari, Mario Latini, Rossana Roila, Giuseppe Lediani, Giovanni Filippini, Giampiero Scortichini, Arianna Piersanti, Elena Rocchegiani, David Ranucci

**Affiliations:** 1 Istituto Zooprofilattico Sperimentale dell’Umbria e delle Marche “Togo Rosati”, 06126 Perugia, Italy; f.barchiesi@izsum.it (F.B.); m.latini@izsum.it (M.L.); g.filippini@izsum.it (G.F.); g.scortichini@izsum.it (G.S.); a.piersanti@izsum.it (A.P.); e.rocchegiani@izsum.it (E.R.); 2 Department of Veterinary Medicine, University of Perugia, Via San Costanzo 4, 06121 Perugia, Italy; rossana.roila@unipg.it (R.R.); david.ranucci@unipg.it (D.R.); 3 Italian Ministry of Health DGSAN Ufficio 2, Viale Giorgio Ribotta, 5, 00144 Roma, Italy; g.lediani@sanita.it

**Keywords:** cadmium, lead, mercury, shellfish, gastropods, sea urchins, dietary intake, dietary exposure

## Abstract

Seafood is a source of nutrients in human diet but also of environmental contaminants and its consumption could pose a risk to consumers’ health. A survey regarding the exposure to cadmium, lead and mercury through the consumption of bivalve mollusks, gastropods and sea urchins collected on Italian coasts was carried out among central Italian population over a period of three years. A limited number of samples exceeds the threshold set by legislation (6 samples) and the average level of contamination was low in all the species considered. The contribution Acceptable Daily Intake (ADI) was higher for cadmium (9.17%) than lead (1.44%) and mercury (0.20%). The benefit-risk evaluation suggests that the bivalve mollusks and sea urchins consumption (Benefit Risk Quotient < 1) could be increased without health detrimental effects.

## 1. Introduction

Heavy metals are known for adverse toxicological effects in humans and food products are considered to be their main source of exposure for general population [1,2,3,4]. Chronic cadmium (Cd) intake is responsible for different organ systems toxicity with reproductive and fertility impairments, skeletal damage, urinary and cardiovascular disorders, central and peripheral nervous deficiency, kidney disease and cancer [5,6,7]. Mercury (Hg) toxicity in nervous, motor, renal, cardiovascular, reproductive, and immune system is reported even at low dose [4,8]. Lead (Pb) is responsible for negative effects on hematopoietic, renal, cardiovascular, reproductive and skeletal systems [3,9,10].

Fish and seafood are regarded as one of the main food sources of these three contaminants as they live in marine environment that could be contaminated by these ubiquitous molecules, which are prone to high distribution in spite of their anthropic or natural origin [11], and they can accumulate Cd, Hg and Pb in their tissue even to a high level [12,13,14].

Maximum limits in various fish and shellfish species are set for these contaminants in different countries [15,16,17,18] and, therefore, monitoring their levels in seafood is of utmost importance [11]. Nonetheless, taking into account the consumers’ habits, a risk based approach to heavy metals exposure has to be considered [2,3,4,19,20,21] for the different seafood available on the market, the ingested dose and the potential beneficial health effects of seafood consumption. Especially, shellfish are considered a valuable source of unsaturated n3 fatty acids such as eicosapentaenoic acid (EPA) and docosahexaenoic acid (DHA) [22,23]. These compounds are proved to exert beneficial effects on human health [24,25], and fish and shellfish consumption could represent a valuable strategy to enhance their dietary intake [26,27,28]. 

The production of bivalve mollusks is considered a sustainable practice as it has a low environmental impact due to limited exploitation of natural resources and to low maintenance costs [29]. Furthermore, Italy is characterized by a wide availability of coastlines which could be more extensively destined to seafood production. Moreover, although the consumption of this products in Italy is already noticeable (17.7% of the economic value of all the fish products consumed in 2018) [30], it could be potentially increased in order to favor the dietary intake of valuable nutrients [31]. In this context a risk-benefit evaluation related to the consumption of seafood, can be considered of utmost importance for the fish sector as well as for consumers public health [32]. 

The aims of this work were the definition of Cd, Hg ad Pb contamination level in marine shellfish, gastropods and sea urchins harvested along the Italian coastline; the assessment of central Italy population exposure; and the benefit-risk evaluation associated to the consumption of these selected products.

## 2. Materials and Methods

### 2.1. Data Source

The analytical results for Cd, Hg and Pb in shellfish (*N* = 2207 after data cleaning) collected along the Italian coastline, from January 2017 until December 2019, were retrieved from SINVSA (Sistema informativo Nazionale Veterinario per la Sicurezza Alimentare), the Platform for Food Safety of the Department for Veterinary Public Health, Nutrition and Food Safety of the Ministry of Health.

SINVSA is a web application, created by the CSN (Centro Servizi Nazionale—Istituto Zooprofilattico Sperimentale dell’Abruzzo e del Molise) and it has been designed to collect information useful for risk assessment in feed and food along the whole production chain, making available all the information related to the industries registry, the official control and the analytical results. It includes all the data on national companies producing food for human consumption and animal feed including the transport and sub-products sector. 

**Figure 1:**
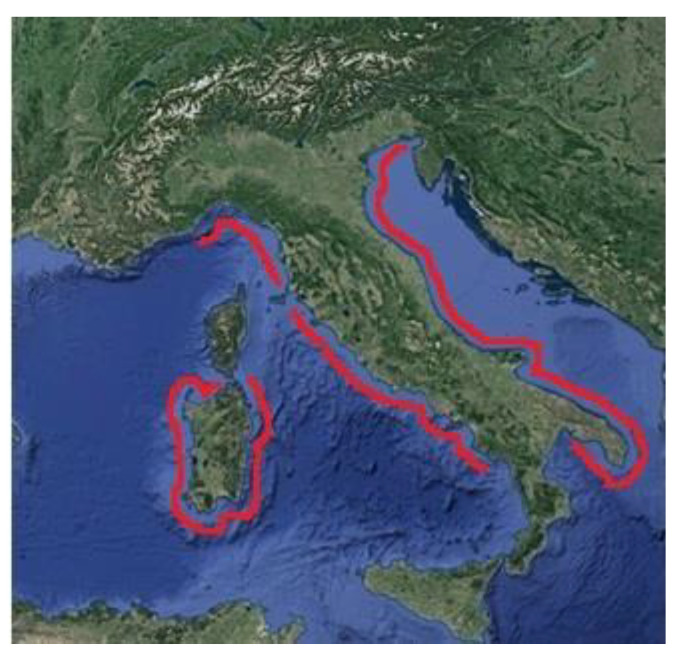
Italian coastline considered for the sampling collection.

The seafood species considered in the survey were bivalve mollusks, gastropods and echinoderms (grouped in classes as reported in Table 1) collected from the coasts of 12 Italian regions (Figure 1).

Data management and descriptive statistical analyses were carried out using Excel datasheet (Microsoft) and Stata 11^®^.

### 2.2. Data Collections

The shellfish analyzed to assess heavy metals contamination were collected by local official competent authority in charge of bivalve mollusks production areas, during classification and monitoring activities compliant to EU regulation [33,34]. Pb, Cd and Hg, were analyzed by official laboratories following UNI CEI EN ISO/IEC 17025 accredited analytical methods [35] and Regulation 333/2007/EC [36] as far as sampling protocols and analytical performances are concerned.

Pb, Cd, Hg were analyzed in 1 g of sample after microwave digestion with 6 mL HNO_3_ (67–69%, *v*/*v*), 2 mL H_2_O_2_ (30%, *v*/*v*), and 100 mL HF (40%, *v*/*v*). 

The appropriately diluted solutions were analyzed by inductively coupled plasma mass spectrometry (ICP-MS) in standard mode using specific mass-to-charge ratios (*m*/*z*) for each element (206 + 207 + 208 Pb, 111 Cd, 202 Hg). Internal standards (i.e., 103Rh) were used to normalize the instrumental response and quantification was matrix-matched. The analytical methods were fully validated in intra-laboratory reproducibility conditions. The LOQs (mg/kg) of the method were: Pb = 0.015, Cd = 0.005, Hg = 0.025. Batch-to-batch precision and accuracy were evaluated by analyzing certified reference materials (Mussel Tissue SRM 2976, NIST Canada).

### 2.3. Dietary Exposure and Risk Characterization

For the definition of contaminants concentration in foodstuff, the left censored data was handled through substitution method. Therefore, when an element concentration was not quantified (<LOQ) its value was assumed to be half of its LOQ according to the middle bound (MB) approach [24,37]. 

The population exposure to Cd, Hg and Pb was assessed by combining seafood classes and contamination results (MB) with specific consumption data, obtained through a detailed questionnaire. Seafood consumption data was derived from a questionnaire administered to 611 residents in central Italy, on both coastal and inland (almost 50 km far from the coast) sites. The participants were 357 females and 253 males; 310 records were obtained from consumers living along the coasts and 301 in the inland. The age of targeted population ranged from 18 to 75 years. The questionnaire was designed to obtain information on the bivalves and echinoderms consumption frequency and consumer’s answers were combined with the food portion size data reported by Italian dietary surveys [38]. The questionnaires were returned anonymously, the participants did not receive any incentives and their consent had been obtained prior to the survey. 

The dietary exposure assessment was conducted as reported by Branciari et al. [39] taking into consideration an average adult weighing 70 kg, all the seafood products and the three target heavy metals.

In order to perform a risk characterization, the results of the exposure assessment were compared to the reference health-based guidance values set for cadmium (Cd = 0.35 ug/kg bw/d) [40], lead (Pb = 0.004 mg/kg bw/d) [41] and mercury (Hg = 0.571 ug/kg bw/d) [42]. This approach allows to carry out a quantitative evaluation of the potentially harmful effects on consumers’ health in relevance to the ingestion of these metals. The results of the risk characterization were expressed as percentage contribution to the Acceptable/Tolerable Daily Intake (ADI/TDI), which represents the amount of a substance in food that can be ingested on a daily basis over a lifetime without a significant health risk [43]. 

### 2.4. EPA and DHA in Seafood and Benefits-Risks Assessment

**Figure 2:**
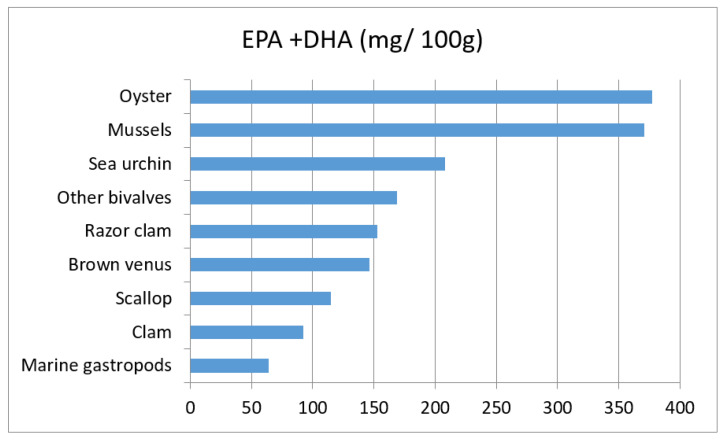
EPA and DHA content in the selected seafood.

Aiming to quantitatively estimate the health benefits of seafood consumption, the EPA and DHA content in mollusks and echinoderms considered, was obtained from literature [32,33,34,35,36,37,38,39,40,41,42,43,44] (Figure 2). The daily dietary intake of such nutrients in the target population was assessed with the same methodology adopted for contaminants. 

Furthermore, to esteem the risks and benefits related to the consumption of the targeted seafood classes, the benefits-risks quotient (BRQ) approach was applied [20]. The benefit of seafood consumption refers principally to the intake of EPA and DHA, recognized as protective factors in cardiovascular diseases and defined as the contribution of the exposure values to the recommended Dietary Reference Intake (RDI) of 250 mg/d for EPA + DHA [24]. Therefore, the contents of the mentioned polyunsaturated fatty acids (PUFA) in the seafood classes considered (Figure 2), were combined with the consumption data. Risk factors were attributed to the ingestion of the targeted metals (Cd, Pb, Hg) which have been proved to be toxic to humans. 

The data was obtained from Prato et al. [32] for oyster, scallop, mussels, brown venus, razor clam, clam and other bivalves, and from Rincón-Cervera et al. [44] for sea urchins and marine gastropods.

*BRQ* values estimate the benfit-risk of the simultaneous ingestion of PUFA and contaminants through seafood species and were calculated according to the following equation [20]:

(1) $$BRQ=\frac{Q_{FA}}{Q_{T}}$$

*Q_FA_* is defined as follows:

(2) $$Q_{FA}=\frac{R_{FA}}{C_{FA}}$$

where *R_FA_* (mg/d) is the recommended daily intake of EPA + DHA (RDI of 250 mg/d for a healthy adult [24] was applied), while *C_FA_* (mg/g) represents the concentration of EPA + DHA in seafood.

The maximum allowable food consumption related to toxic effects (*Q*_T_) can be calculated according to the following equation:

(3) $$Q_{T\;\;}=\frac{RfD*BW}{c}$$

where *RfD* (mg/kg bw/d) is the reference dose of a pollutant defined through the ADI/TDI of each contaminant considered, *BW* is the standard bodyweight set, as mentioned above, at 70 kg, and *c* (mg/g) is the concentration of each toxic molecule in the targeted food products. 

*BRQ* values below 1 suggest that achieving the recommended intake of EPA + DHA poses no evident risk to human health related to the simultaneous intake of the pollutant through seafood consumption [19,20].

## 3. Results and Discussion

The results of Cd, Hg and Pb in shellfish, gastropods and echinoderms for the three-year survey are presented in Table 2, Table 3 and Table 4. For Pb the concentration was always under the maximum limits set by EU Regulation (MRL= 1.5 mg/kg) [16], for Hg 1 sample (1 sea urchin in 2017) exceeds the maximum (MRL= 0.50 mg/kg) and for Cd 5 samples (1 mussel in 2017 and 4 gastropods in 2018) exceed maximum level (MRL= 1.0 mg/kg). Scallops and brown venus samples were always above LOQ for Cd, nonetheless, the other classes have only few samples below LOQ. Cadmium levels were higher in oysters (average middle bound MB = 0.218 mg/kg) and gastropods (MB = 0.217 mg/kg) followed by scallops (MB = 0.117 mg/kg). These results may be explained in respect to the different filtering capacity of the species, the specific living environment and, therefore, their accumulation abilities [45,46]. The values recorded in the present survey are similar to those referred for shellfish by other authors [43,47,48], even though other shellfish species, collected in different environmental conditions, showed higher levels on specific sites [49]. However, higher Cd values are registered in gastropods and oyster [44,48]. A relevant factor influencing the bivalves capacity in accumulating Cd, particularly oysters, is their position in the water column. Indeed oysters, growing at the bottom, can accumulate Cd up to 10 times higher than oysters growing in the same site, in baskets placed in the surface of the water [45]. As reported in literature, Cd concentration tends to be higher in deeper waters and decreases in surface water [50]. Concerning gastropods, the possible factors implicated in Cd accumulation could be their living environment (they generally live buried in fine sediments [51]) and the presence of Cd-binding proteins (metallothioneins) in their body, which are involved in shell formation [52,53].

Regarding Hg, the number of samples above LOQ was lower than those recorded for Cd and Pb; the number of above LOQ samples for Hg recorded in gastropods and brown venus was higher than in the other species analyzed. Samples of razor clam revealed the highest level of Hg contamination (an average of MB = 0.087 mg/kg), followed by gastropods and mussels (an average of MB = 0.036 and 0.024 mg/kg, respectively).

The data is compliant to that reported in literature and show a relatively low Hg contamination in shellfish [32,45,49,54]. It is well known that Hg, as a result of its bioaccumulation and biomagnification capacity in marine environment, tends to reach higher levels in predator fish which are the most relevant food exposure source to humans [4,54,55,56]. 

The selected seafood always shows a prevalence of samples above LOQ close to 100% for Pb. The highest average bivalve mollusks values were detected in sea urchins (0.203 mg/kg) followed by scallops (0.191 mg/kg) and mussels (0.174 mg/kg). Similar results are reported for different shellfish harvested in the north Adriatic Sea [32,45] and in the South of Spain [47], but they are higher than on some specific sites on the East African coast (i.e., the Gulf of Suez) [50]. Even echinoderm can accumulate Pb and other heavy metals present in the marine environment [57]. Among echinoderms, the purple sea urchin is considered a bio-indicator for the monitoring of metal pollution along the Mediterranean and Atlantic coasts [58,59] as it is able to concentrate the pollutants to a greater extent than all the other shellfish. Consequently, sea urchins remarkably contribute to transfer heavy metals and other pollutants to higher trophic levels [60]. 

**Figure 3:**
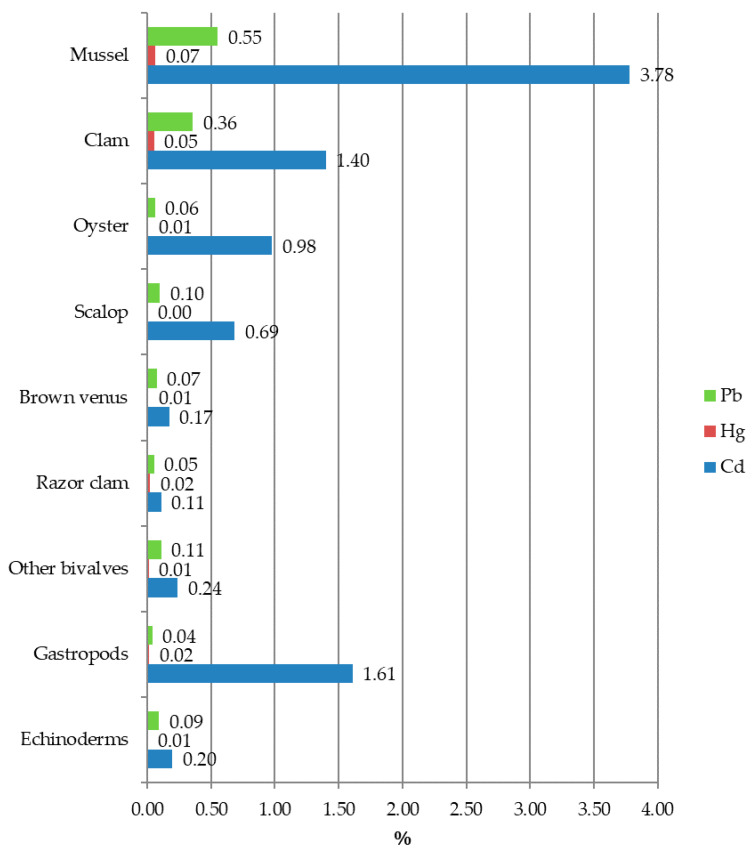
Contribution to the metals ADIs (%) of the selected seafood.

As far as the risk characterization is concerned, the contribution to ADI of the various shellfish studied is reported in Figure 3. The average contribution of each product to ADI of the population considered was extremely low for Hg and Pb, with values always below 1%, but higher for Cd, with values not exceeding 4%. The contribution to ADI for the three metals was higher for mussels followed by clams, oysters and scallops. Regarding Cd, a contribution to ADI of 2% was recorded in gastropods: this value is relatively low, but it is higher than for shellfish and echinoderms. The consumption frequency and the portion size surely affect these results: mussels are the most frequently eaten shellfish included in the present survey (average consumption of 8.88 g/kg bw/die for mussels and 9.12 g/kg bw/die for clams, respect to 1.09 g/kg bw/die for oysters, 1.44 g/kg bw/die for scallops, 1.27 g/kg bw/die for brown venus, 1.44 g/kg bw/die for razor clams and other bivalves 2.56 mg/kg bw/die).

The contribution to ADI by sea urchins is limited as a result of a very low consumption (1.25 g/kg bw/die). On the other hand, gastropods, although modestly consumed (1.82 g/kg bw/die), contribute to Cd ADI to a higher extent than other species considered, due to their accumulation capacity. The contribution of each species to ADI provides a measure of safety during long-term exposure upon consumption [43], therefore, the reported results (Figure 3) suggest a negligible public health risk of exposure to metals through the consumption of the seafood species taken into consideration. These results are in accordance with other authors [57] who state that there is no significant health risk of humans’ exposure to Cd, Hg and Pb upon consumption of shellfish. Furthermore, the bio accessibility for metals like cadmium in cooked shellfish is reduced and thus further mitigates health risk [61]. 

**Figure 4:**
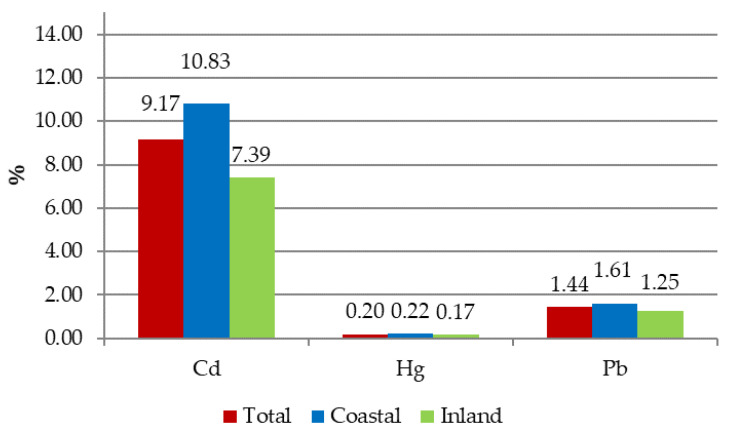
ADI contribution (%) in relation to geographical distribution of population.

The overall mean contribution to the ADI of the three targeted metals upon the above seafood species consumption, dividing the population in respect to their geographical distribution (inland or coastal), is reported in Figure 4.

The results confirm a higher contribution to the reference value (ADI) in case of Cd in comparison to that of Pb and Hg. The Cd contribution to ADI registered in coastal consumers was greater, probably due to a higher seafood consumption. This uneven contribution was not observed in the other heavy metals subject of the present study (Figure 4). 

In this research, the benefit-risk quotient was applied in order to evaluate the simultaneous effect on human health of EPA and DHA ingestion and metal contaminants present in seafood products. As shown in Table 5, the *BRQ* for most of the groups of seafood analyzed was <1, ranging between 0.00 and 0.57. This result implies that healthy consumers potentially eating enough sea products to achieve the RDI for EPA + DHA, would not be exposed to an increased health risk due to the simultaneous exposure to the toxic metals analyzed.

The unique exception to this pattern is gastropods, registering a BRQ of 3.46 for Cd, meaning that for this seafood species the risk associated with the exposure to this metal prevails over the benefits of polyunsaturated fatty acids intake. In spite of the moderate consumption of gastropods registered by the questioned population, this outcome is likely due to the combination of the low content of EPA+DHA (Table 1) and the relatively high concentration of metals associated with these marine species, due to their major route of trace metal uptake tracking [62]. 

However, as reported by other authors in different environments, these results confirm that the benefits of sea products intake should outweigh the associated risks, when considering the average healthy population [57].

## 4. Conclusions

The average levels of Cd, Hg and Pb detected in mollusks and sea urchins from the Italian coastline are low and, therefore, the exposure of the targeted adult population to these metals is moderate, even when higher shellfish portions are consumed, as it is the habit of the coastal population. Benefit-risk evaluation revealed that the frequency of the above seafood consumption could be enhanced with the aim to increase EPA + DHA intake, without adverse effects.

## Figures and Tables

**Table 1:** Seafood classes and species considered.

Classes	Species Scientific Name
**Bivalve mollusks**	
Mussel	*Mytilus galloprovincialis*
	*Mytilus edulis*
	*Modiolus barbatus*
Clam	*Ruditapes decussatus*
	*Ruditapes phylippinarum*
	*Chamelea gallina*
	*Venus verrucosa*
Oyster	*Ostrea edulis*
	*Crassostrea gigas*
	*Crassostrea angulata*
Scallop	*Flexopecten glaber*
	*Pecten spp.*
	*Mimachlamys varia*
	*Chlamys spp.*
Brown venus	*Callista chione*
Razor clam	*Solen siliqua*
Other bivalves	*Arca noae*
	*Cardium edule*
	*Cerastoderma spp.*
	*Donax trunculus*
**Marine Gastropods**	
Gastropods	*Hexaplex trunculus*
	*Nassarius mutabilis*
	*Muricidae*
	*Bolinus brandaris*
	*Buccinum undatum*
**Echinoderms**	
Sea urchins	*Paracentrotus lividus*

**Table 2:** Cadmium (Cd) levels (mg/kg) in selected seafood in the three-year period.

Year	Classes	Analyzed Samples	Above LOQ Samples (%)	Min	Max	Average (MB) ^1^
2017	Mussel	152	137/(90)	0.030	1.150	0.099
	Clam	260	226/(87)	0.010	0.140	0.035
	Oyster	7	7/(100)	0.130	0.530	0.240
	Scallop	37	37/(100)	0.050	0.370	0.100
	Brown venus	36	36/(100)	0.010	0.060	0.030
	Razor clam	1	1/(100)	0.003		0.003
	Other bivalves	28	9/(32)	0.010	0.270	0.024
	Gastropods	59	56/(95)	0.010	0.770	0.228
	Echinoderms	1	0/(0)			0.003
2018	Mussel	301	287/(95)	0.010	0.880	0.105
	Clam	308	267/(87)	0.010	0.170	0.044
	Oyster	47	46/(98)	0.070	0.840	0.235
	Scallop	38	38/(100)	0.050	0.400	0.110
	Brown venus	36	36/(100)	0.010	0.080	0.040
	Razor clam	16	16/(100)	0.010	0.060	0.020
	Other bivalves	30	6/(20)	0.010	0.350	0.028
	Gastropods	44	43/(98)	0.010	1.880	0.303
	Echinoderms	9	9/(100)	0.020	0.300	0.060
2019	Mussel	265	239/(90)	0.020	1.000	0.108
	Clam	276	232/(84)	0.010	0.180	0.034
	Oyster	40	38/(95)	0.060	0.880	0.181
	Scallop	25	25/(100)	0.060	0.400	0.140
	Brown venus	26	26/(100)	0.020	0.070	0.030
	Razor clam	25	20/(80)	0.010	0.340	0.033
	Other bivalves	24	2/(8)	0.010	0.280	0.014
	Gastropods	17	17/(100)	0.010	0.470	0.120
	Echinoderms	8	8/(100)	0.020	0.080	0.050

^1^ MB = middle bound.

**Table 3:** Mercury (Hg) levels (mg/kg) in selected seafood over the three-year period.

Year	Classes	Analyzed Samples	Above LOQ Samples (%)	Min	Max	Average(MB) ^1^
2017	Mussel	185	71/(38)	0.030	0.300	0.038
	Clam	260	64/(25)	0.030	0.140	0.024
	Oyster	7	2/(29)	0.030	0.110	0.029
	Scallop	37	0/(0)			0.013
	Brown venus	36	13/(36)	0.030	0.070	0.022
	Razor clam	1	0/(0)			0.010
	Other bivalves	28	9/(32)	0.030	0.170	0.028
	Gastropods	59	51/(86)	0.030	0.100	0.045
	Echinoderms	55	1/(20)	0.063		0.024
2018	Mussel	302	104/(34)	0.030	0.190	0.032
	Clam	308	89/(29)	0.030	0.180	0.023
	Oyster	47	17/(36)	0.030	0.260	0.026
	Scallop	38	0/(0)			0.013
	Brown venus	36	27/(75)	0.030	0.090	0.041
	Razor clam	16	13/(81)	0.070	0.210	0.108
	Other bivalves	29	1/(3)	0.040		0.013
	Gastropods	44	34/(77)	0.030	0.100	0.034
	Echinoderms	10	1/(10)	0.040		0.015
2019	Mussel	265	34/(13)	0.030	0.170	0.017
	Clam	276	70/(25)	0.030	0.130	0.022
	Oyster	40	7/(18)	0.030	0.110	0.019
	Scallop	25	0/(0)			0.013
	Brown venus	26	19/(73)	0.030	0.060	0.033
	Razor clam	25	14/(56)	0.700	0.230	0.084
	Other bivalves	24	1/(4)	0.040		0.014
	Gastropods	17	8/(47)	0.030	0.100	0.030
	Echinoderms	10	2/(20)	0.030	0.040	0.018

^1^ MB = middle bound.

**Table 4:** Lead (Pb) levels (mg/kg) in selected seafood over the three-year period.

Year	Classes	Analyzed Samples	Above LOQ Samples (%)	Min	Max	Average(MB) ^1^
2017	Mussel	152	138/(91)	0.040	0.560	0.128
	Clam	260	223/(86)	0.020	0.350	0.078
	Oyster	7	7/(100)	0.090	0.400	0.190
	Scallop	37	37/(100)	0.020	0.500	0.150
	Brown venus	36	36/(100)	0.030	0.260	0.110
	Razor clam	1	0			0.008
	Other bivalves	28	25/(89)	0.030	0.450	0.117
	Gastropods	59	44/(75)	0.020	0.170	0.032
	Echinoderms	4	4/(100)	0.050	0.370	0.190
2018	Mussel	302	285/(94)	0.020	0.740	0.180
	Clam	308	267/(87)	0.020	0.740	0.114
	Oyster	47	47/(100)	0.030	0.780	0.150
	Scallop	38	37/(97)	0.030	0.420	0.175
	Brown venus	36	36/(100)	0.050	0.360	0.150
	Razor clam	16	16/(100)	0.040	0.180	0.100
	Other bivalves	29	27/(93)	0.030	0.260	0.103
	Gastropods	44	37/(84)	0.020	0.410	0.052
	Echinoderms	10	10/(100)	0.040	0.400	0.200
2019	Mussel	265	236/(89)	0.020	1.070	0.215
	Clam	276	232/(84)	0.020	1.080	0.136
	Oyster	40	38/(95)	0.030	0.370	0.124
	Scallop	18	16/(89)	0.020	0.660	0.250
	Brown venus	26	26/(100)	0.070	0.430	0.230
	Razor clam	25	24/(96)	0.070	0.590	0.212
	Other bivalves	24	24/(100)	0.040	0.530	0.130
	Gastropods	17	16/(94)	0.020	0.310	0.104
	Echinoderms	10	10/(100)	0.020	0.520	0.220

^1^ MB = middle bound.

**Table 5:** BRQ for Cd, Hg and Pb in the selected seafood.

	Cd	Hg	Pb
Mussel	0.33	0.01	0.05
Clam	0.31	0.01	0.08
Oyster	0.57	0.00	0.04
Scallop	0.33	0.00	0.05
Brown venus	0.23	0.01	0.10
Razor clam	0.13	0.03	0.07
Other bivalves	0.13	0.00	0.00
Gastropods	3.46	0.00	0.00
Echinoderms	0.12	0.00	0.06
